# Supplementary material for: Real-time observation of DNA recognition and rejection by the RNA-guided endonuclease Cas9
Source: Nat Commun. 2016 Sep 14;7:12778. doi: 10.1038/ncomms12778 (PMC5027287; doi:10.1038/ncomms12778)
Supplement: Supplementary Information — Supplementary Figures 1-13, Supplementary Tables 1-2 and Supplementary References [file ncomms12778-s1.pdf]

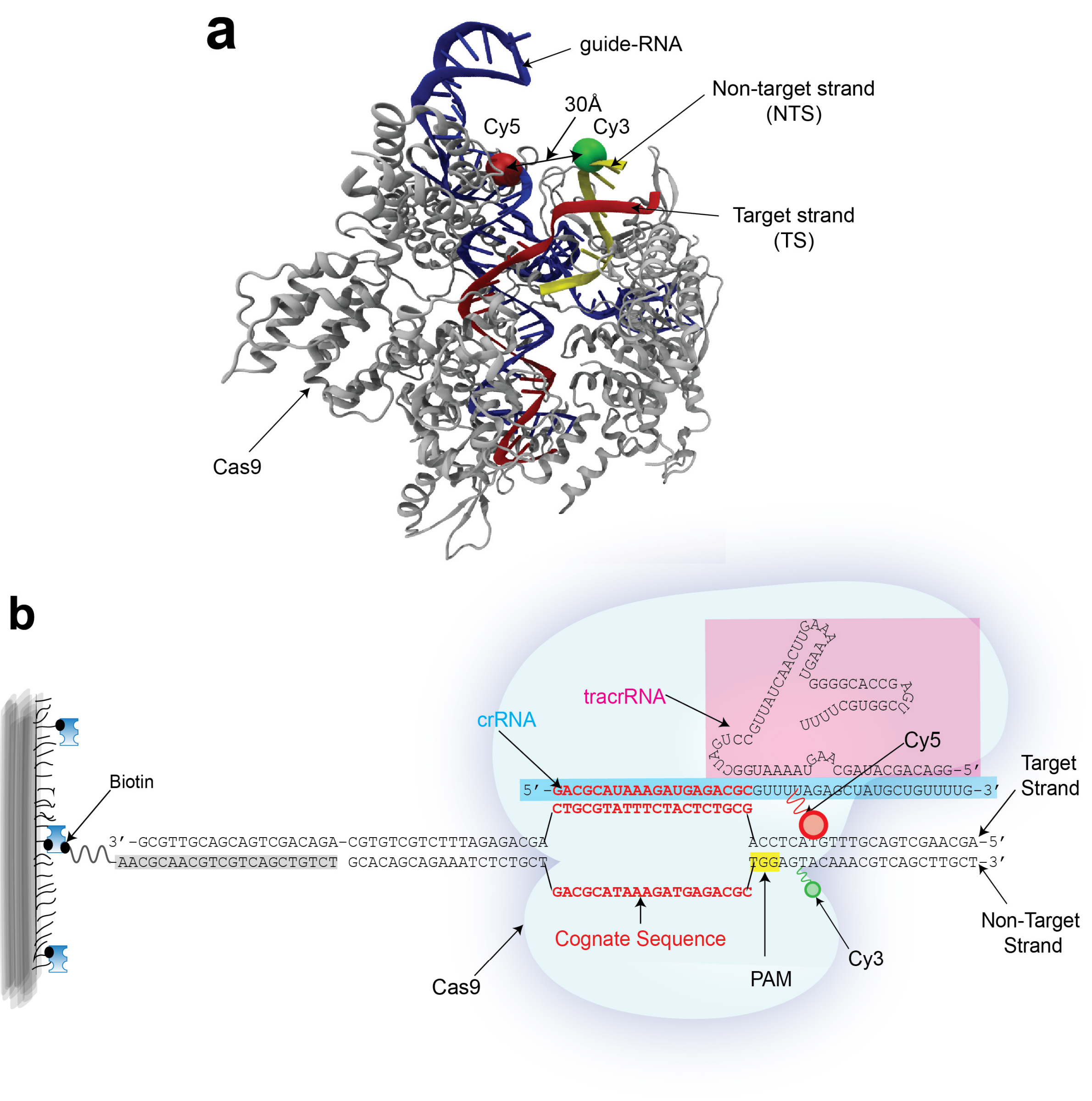

Supplementary Figure 1. FRET probe labeling locations in the Cas9-RNA-DNA complex. (a) Cy3 and Cy5 labeling locations shown in the crystal structure of Cas9-RNA bound to a cognate DNA target (PDB ID: 4UN3)[1]. The strand hybridized with the guide RNA to form the RNA-DNA heteroduplex is referred to as the target strand while the other strand, containing the PAM (5'-NGG-3'), is the non-target strand. **(b)** Schematic of a bound Cas9-RNA-DNA complex showing the base pairing between different components. The sequences shown in red denote the

cognate sequence of the DNA target and the complementary guide sequence of the crRNA. The DNA sequence highlighted in light gray is a separate 22 nucleotide-long biotinylated adaptor used for surface immobilization of DNA target molecules.

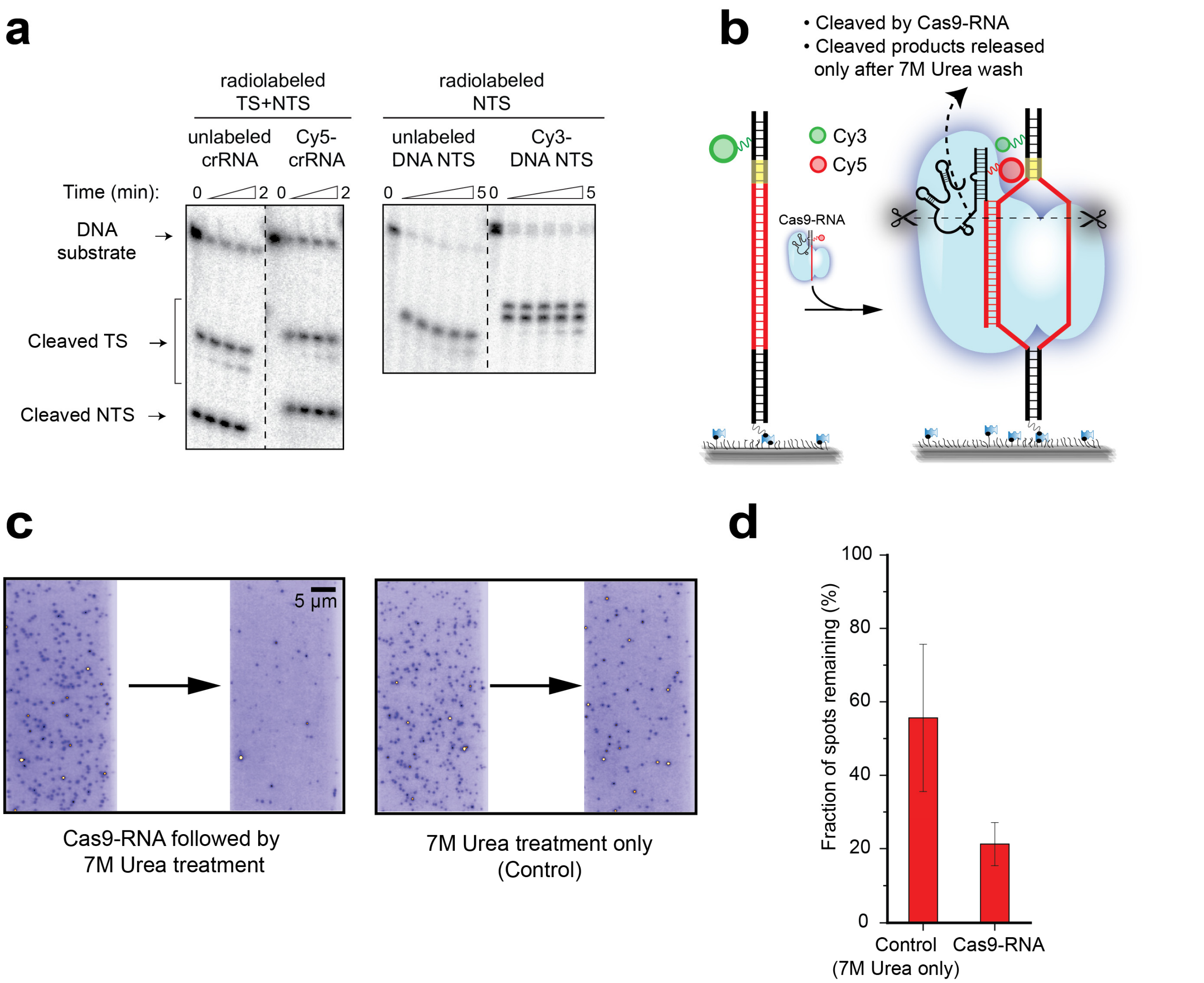

Supplementary Figure 2. Target cleavage activity is not impaired by fluorescent labeling of guide RNA or DNA target.

(a) DNA cleavage assays using fluorescently labeled components, analyzed by denaturing PAGE. TS, target strand; NTS, non-target strand. Note that Cy3-DNA exhibits retarded gel mobility compared to unlabeled DNA, giving rise to two cleavage product bands and two substrate bands due to incomplete labeling. **(b)** Single-molecule assay to monitor DNA cleavage of the cognate DNA target. Because Cas9-RNA retains high-affinity binding to DNA cleavage products, 7 M urea was required to release Cas9-RNA and the DNA product containing Cy3[2]. **(c-d)** Disappearance of Cy3 spots resulted from Cas9-RNA mediated DNA cleavage, as shown by **(c)** fluorescence images and **(d)** quantification of Cy3 spot counts. About 20% of spots

remained after Cas9-RNA reaction followed by urea wash compared to more than 50 % in the control with urea treatment only. [Cas9-RNA] = 1 μM. Error bars represent s.d. for $n = 3$.

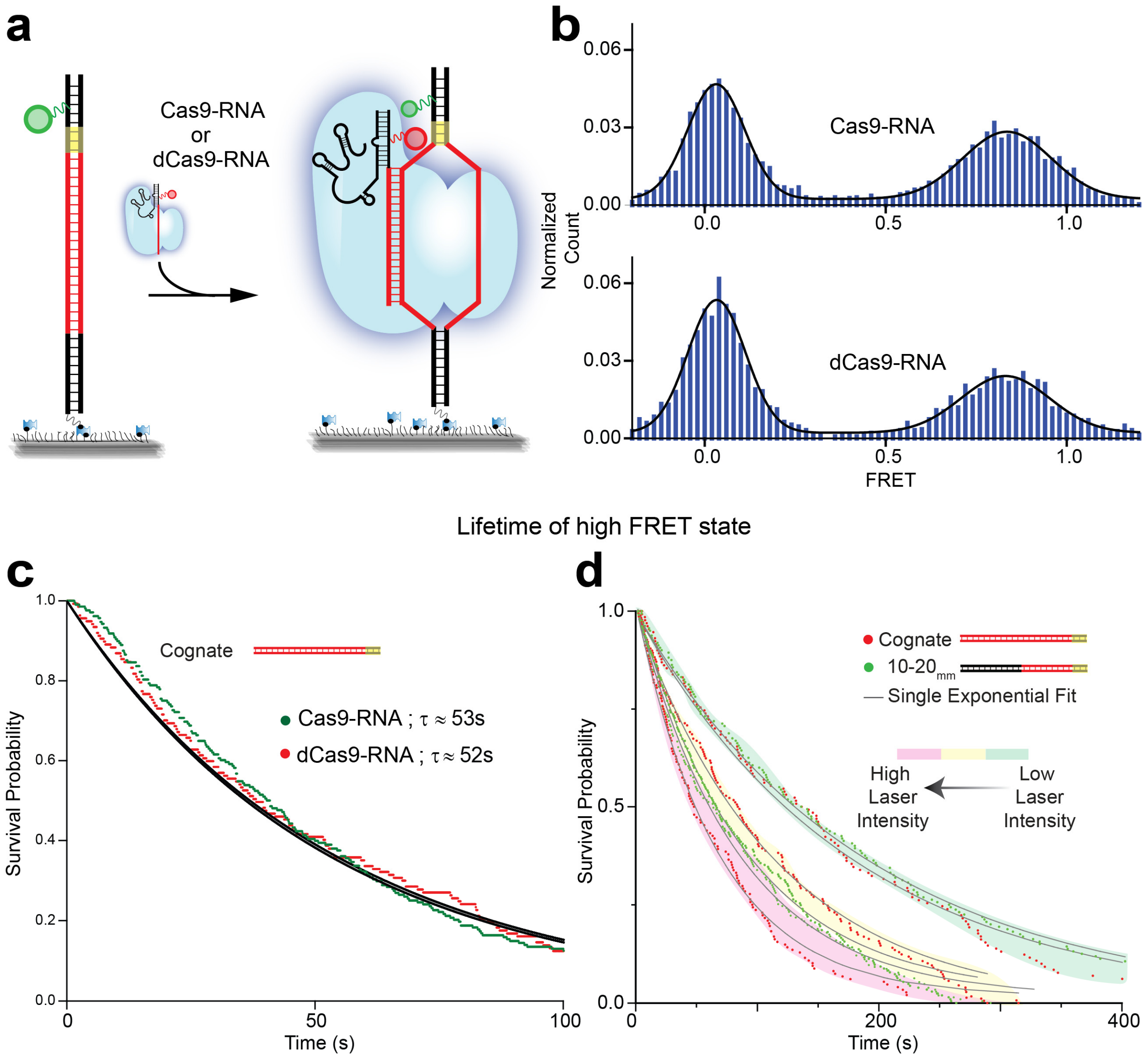

Supplementary Figure 3. Comparison between Cas9 and dCas9, and high stability of Cas9-RNA-DNA for certain DNA targets.

(a) Cas9 or dCas9 were assembled with acceptor-labeled guide RNA to monitor binding to a donor-labeled cognate DNA target. **(b-c)** Cas9 and dCas9 were indistinguishable in our binding assay, as shown by the similarity of (**b**) FRET histograms at 20 nM Cas9-RNA/dCas9-RNA and (**c**) lifetimes of the high FRET state at 10 nM Cas9-RNA/dCas9-RNA, fit with a single exponential decay. The number of molecules used for the FRET histograms was 346 (Cas9-RNA) and 297 (dCas9-RNA). **(d)** DNA targets with contiguous complementarity larger than 8

bps in the PAM-proximal end of the protospacer showed long-lived binding as shown in (**c**). To confirm that the slow decay was caused by photobleaching instead of Cas9-RNA dissociation, we monitored the loss of signal at different laser intensities. As expected, the apparent lifetime of the high FRET state increased with decreasing laser intensity, reaching 3.3 min for the lowest intensity tested. [Cas9-RNA] = 20nM.

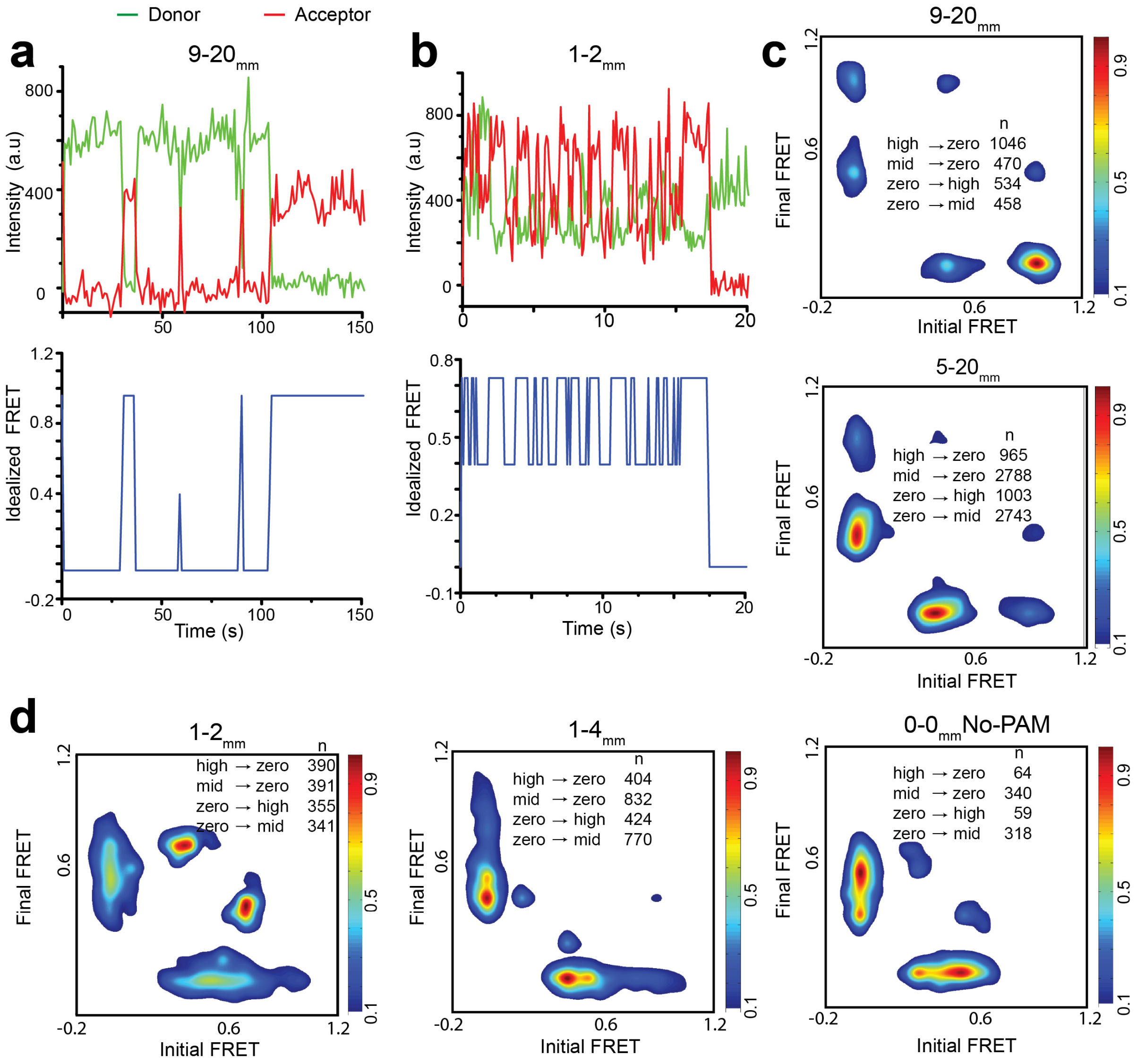

Supplementary Figure 4. Hidden Markov model analysis and transition density plots reveal transitions between three different FRET states.

(a-b) Representative single-molecule fluorescence time trajectories and their idealized FRET time trajectories, obtained using hidden Markov modeling[3], for (**a**) the 9-20_{mm} DNA target and (**b**) the 1-2_{mm} DNA target. [Cas9-RNA] = 20 nM. **(c-d)** Transition density plots (TDP) reveal the relative frequencies of transitions between the initial FRET value and the final FRET value, as identified by hidden Markov modeling. The heat maps are separately scaled for each DNA

target. A small proportion of rapid FRET fluctuations between high and mid FRET states were also observed. The relative population of transitions to/from the mid FRET state increased with increasing mismatches at both **(c)** PAM-distal and **(d)** PAM-proximal ends. The actual number of transitions from high to zero FRET state and mid to zero FRET state are shown within TDPs.

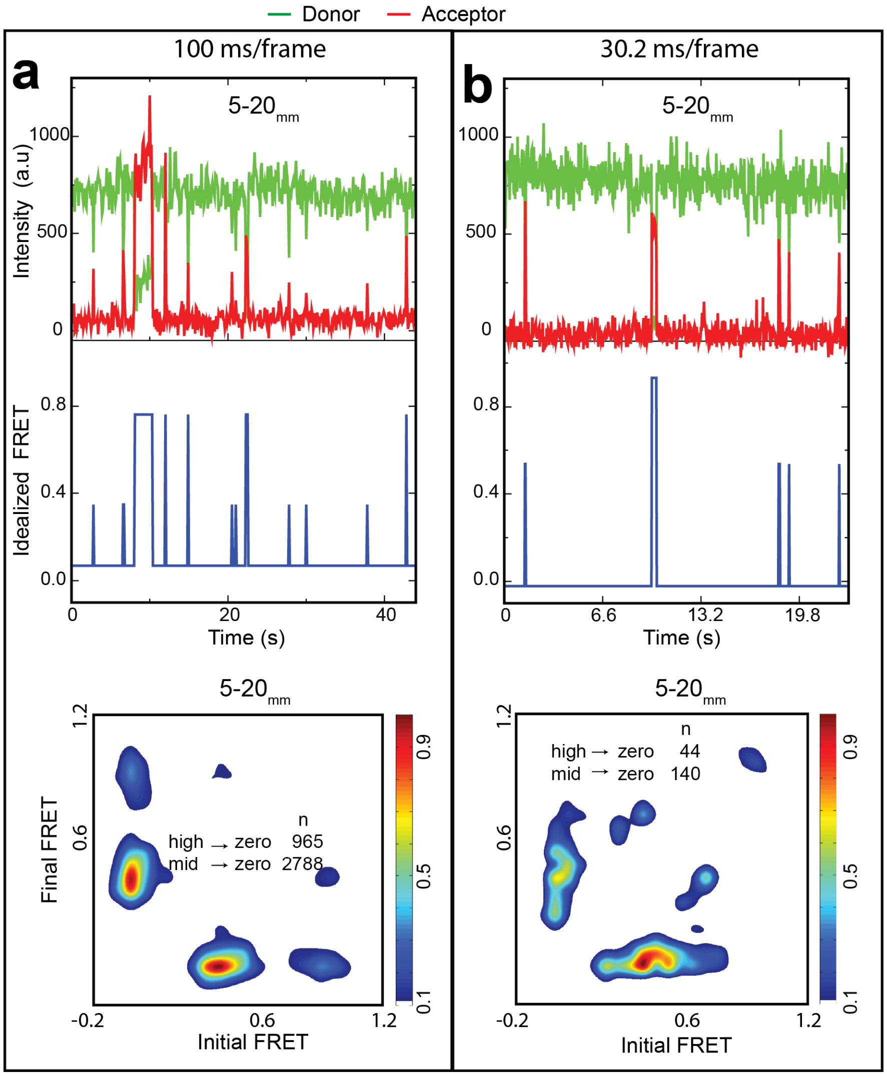

Supplementary Figure 5. Transition density plots for 5-20$_{mm}$ DNA target at two different frame rates of image acquisition.

In order to test if the short-lived mid FRET state observed at 100 ms time resolution was affected by time resolution, we performed additional measurements at 30.2 ms time resolution and compared the results for 5-20$_{mm}$ and found no appreciable difference. **(a)** A representative single-molecule fluorescence time trajectory showing Cas9-RNA binding at 100 ms/frame (top) and the associated transition density plot (bottom). [Cas9-RNA] = 20 nM. **(b)** A representative

single-molecule fluorescence time trajectory showing Cas9-RNA binding at 30.2 ms/frame (top) and the associated transition density plot (bottom). [Cas9-RNA] = 20 nM.

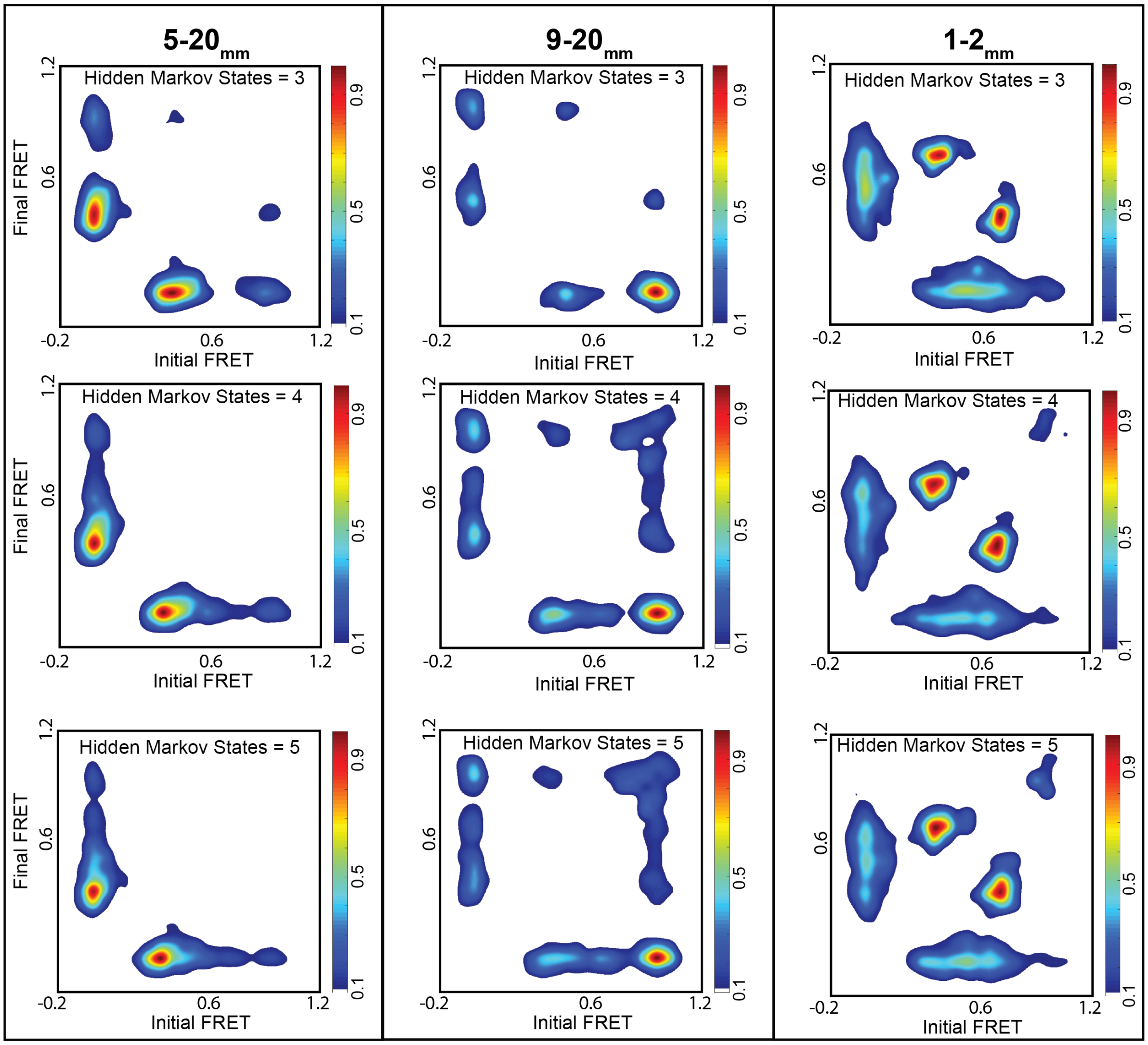

Supplementary Figure 6. Transition density plots for 9-20$_{mm}$, 5-20$_{mm}$ and 1-2$_{mm}$ DNA targets with different inputs for hidden Markov modeling.

3 hidden Markov states were sufficient to capture the different FRET states of Cas9 targeting as any additional input state for hidden Markov modeling did not, evidently, result in any new discrete FRET state.

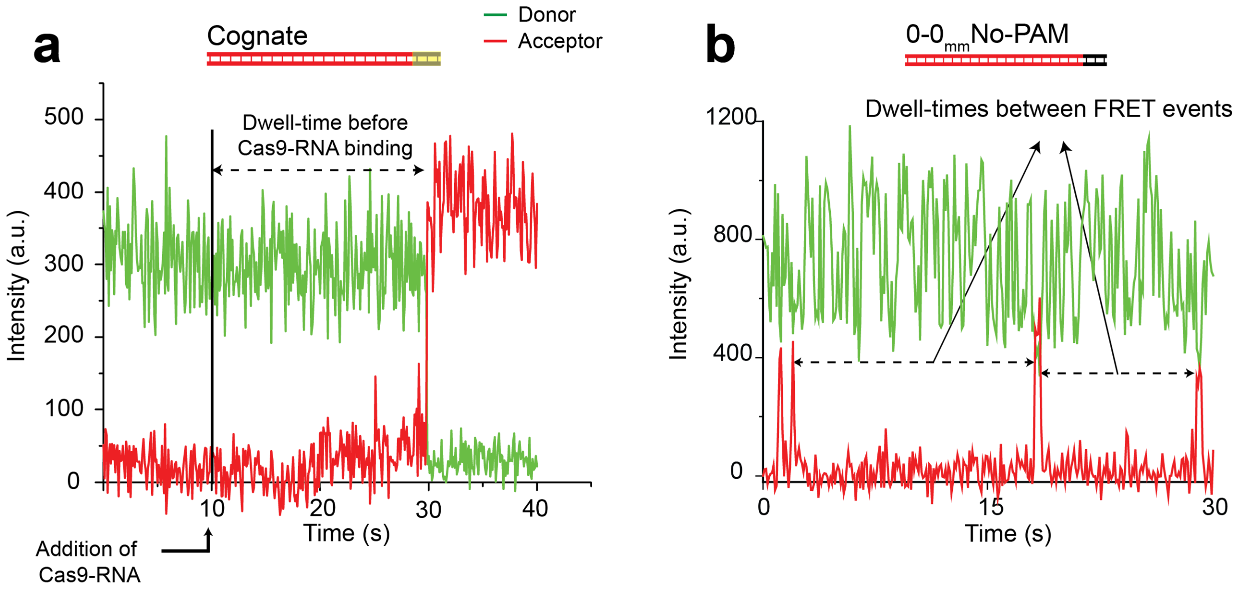

Supplementary Figure 7. Determination of dwell times in the unbound state.

(a) A representative smFRET time trajectory shows Cas9-RNA binding the cognate DNA target in real time. A 20 nM Cas9-RNA solution was added at the indicated time point and the dwell time in the zero FRET unbound state until the appearance of FRET was recorded. **(b)** A representative smFRET time trajectory for a DNA target with full sequence complementarity to guide RNA but without a PAM motif, obtained by imaging under steady state conditions with 20 nM Cas9-RNA in solution. Dwell times in the zero FRET unbound state between upward spikes in FRET due to transient binding events were recorded. See Supplementary Figure 7 for the subsequent analysis.

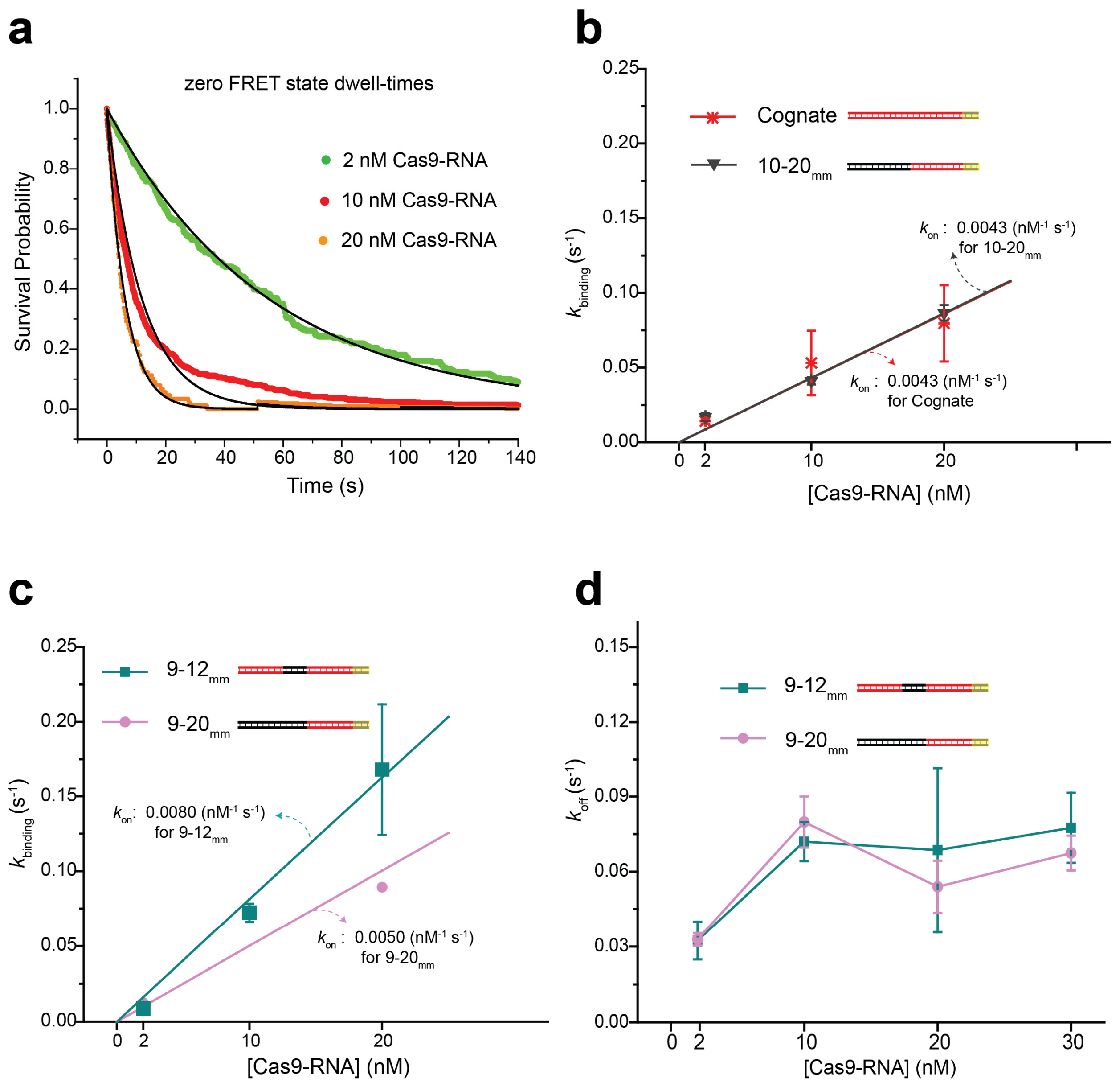

Supplementary Figure 8. Determination of the rates of FRET appearance and disappearance.

(a) Survival probability of dwell times of zero FRET state for 9-12$_{mm}$ DNA target (colored dots) fit with a single exponential decay (black line), to determine the observed binding rate, $k_{binding}$ at three different Cas9-RNA concentrations. **(b, c)** $k_{binding}$ vs. [Cas9-RNA] for different DNA

constructs as noted. Linear fits were used to estimate the bimolecular association constant (k_{on}).

(d) k_{off} vs. [Cas9-RNA]. Error bars represent s.d. for $n = 3$ ($n = 2$ for few sets).

a

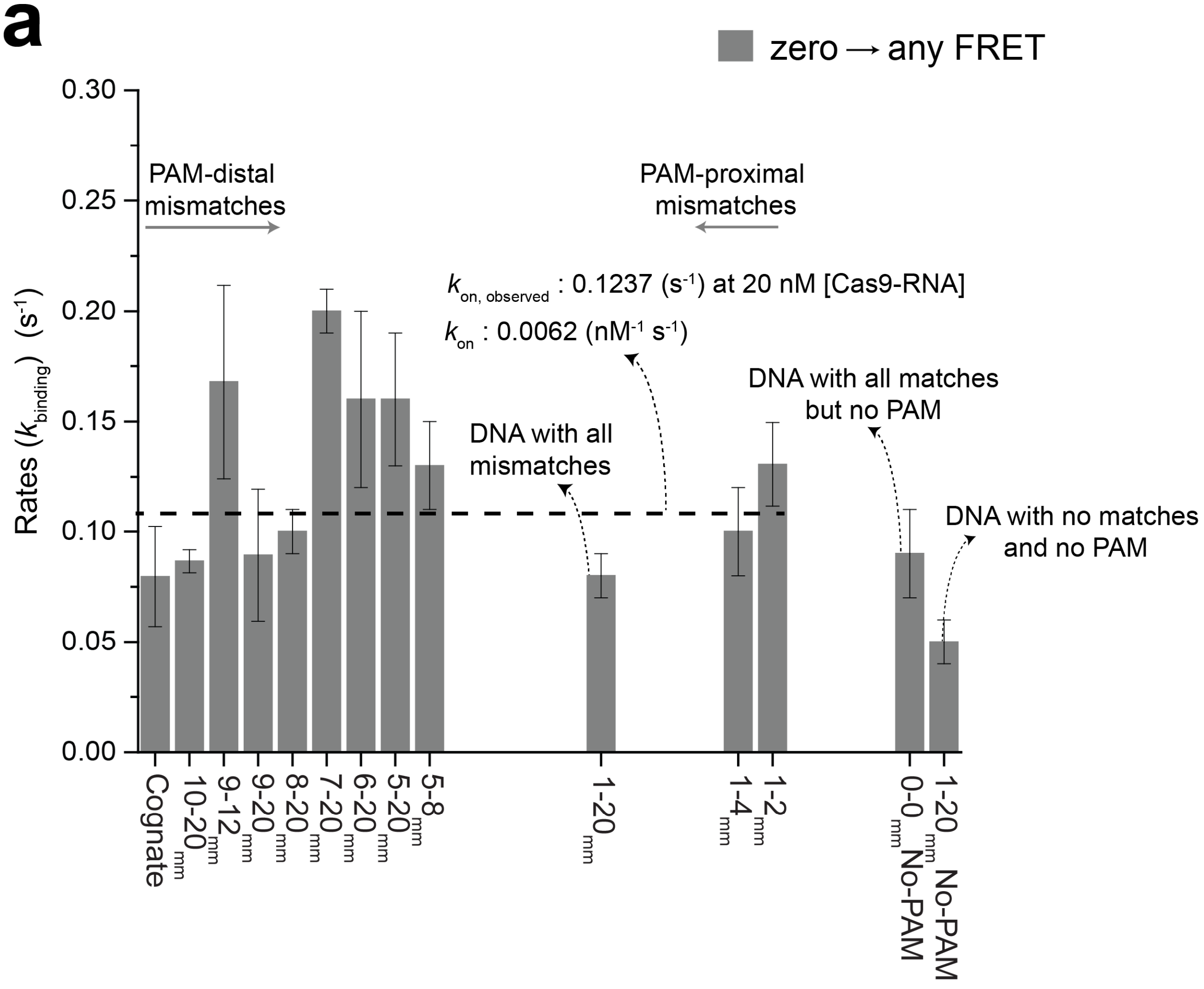

Supplementary Figure 9. The binding rates.

(a) Rate of Cas9-RNA DNA binding for a panel of DNA targets. For these calculations of the binding rate, $k_{binding}$, both the mid and high FRET state were taken together as a single state. A straight line fit to the observed binding rates was used to estimate the binding constant (k_{on}) of Cas9-RNA averaged over all the DNA targets with PAM. [Cas9-RNA] = 20 nM. Error bars represent s.d. for n = 3 (n = 2 for few sets).

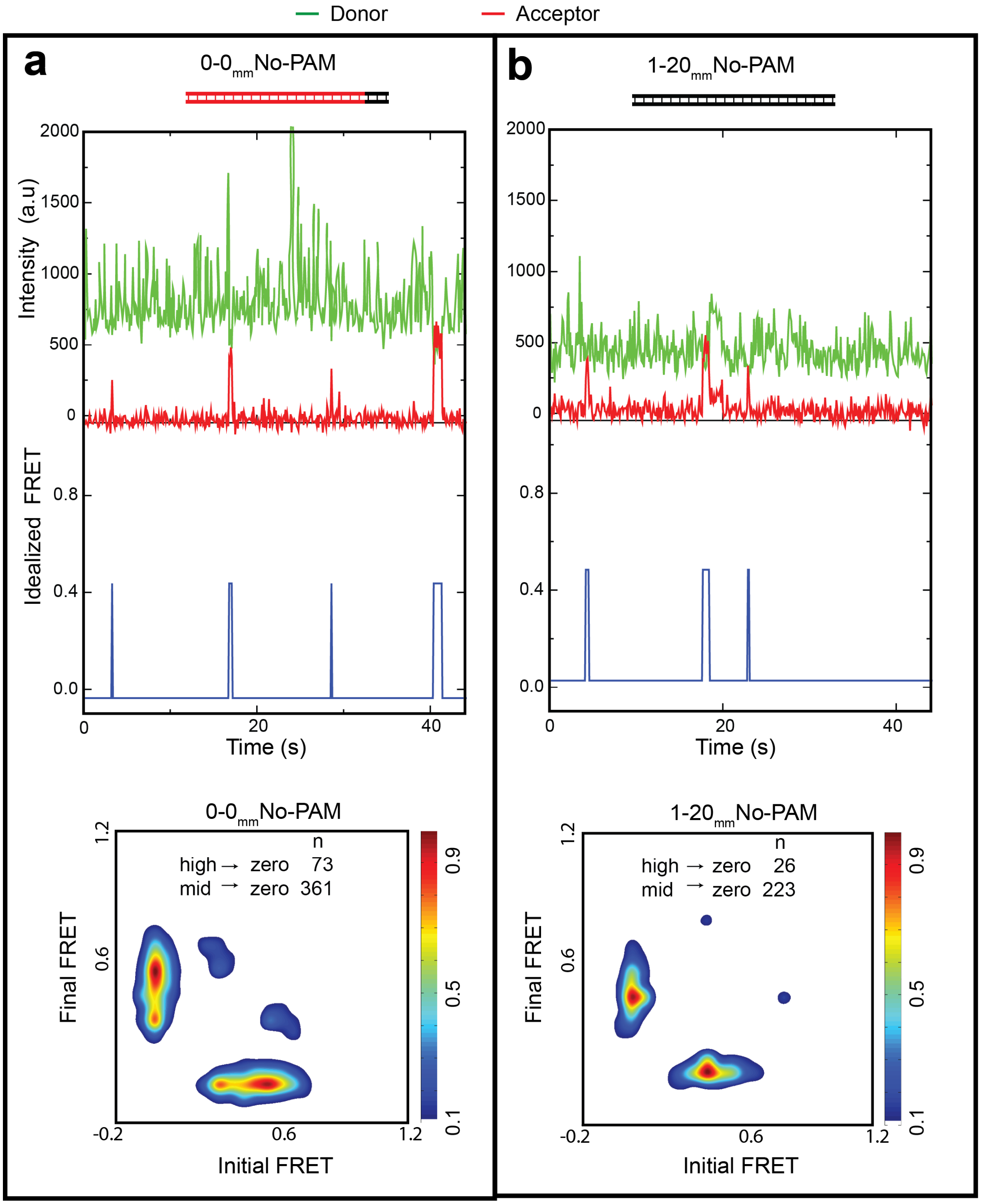

Supplementary Figure 10. Binding dynamics for 'PAM-less' cognate DNA target and non-cognate target with PAM.

Representative single-molecule fluorescence time trajectories (top) and associated transition

density plots (bottom) of DNA target with full sequence complementarity (cognate) to guide-RNA but lacking a PAM motif (**a**, 0-0_{mm}No-PAM) and with no matches and no PAM (**b**, 1-20_{mm}No-PAM). [Cas9-RNA] = 20 nM. The number of transitions from high to zero FRET states and mid to zero FRET states are indicated within the plots.

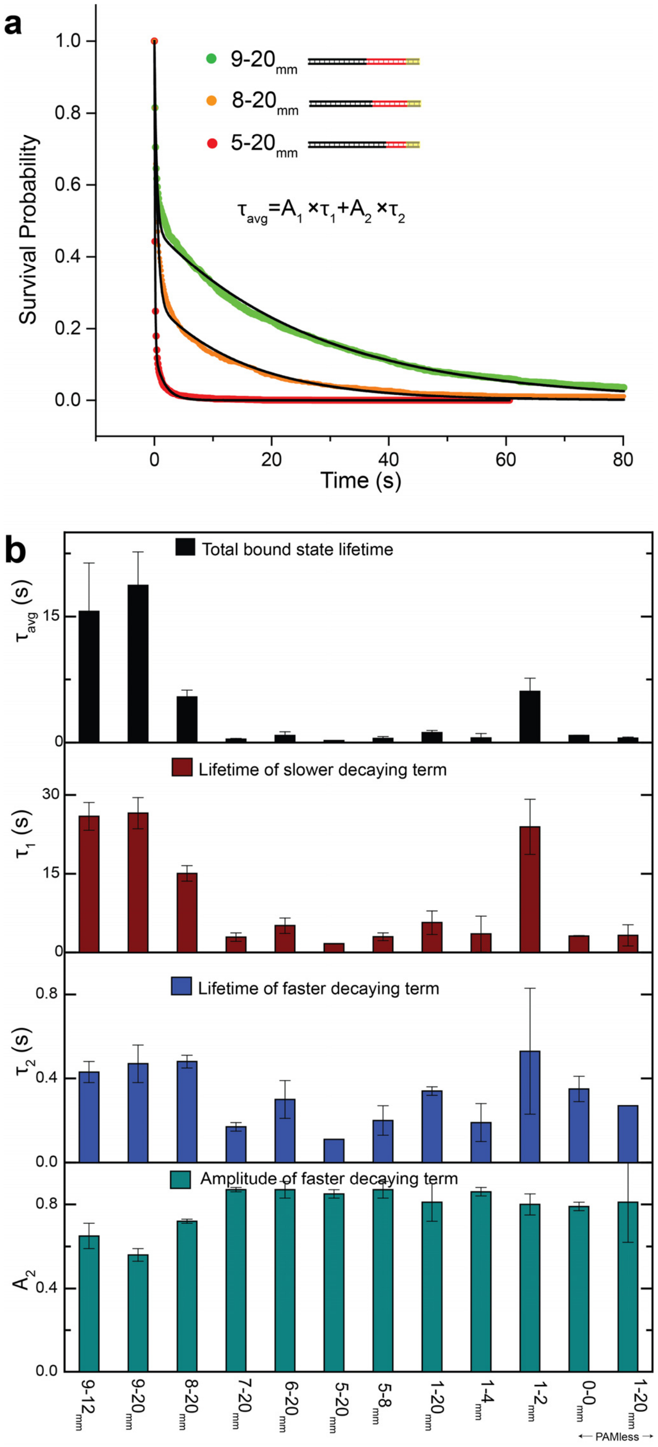

Supplementary Figure 11. Fitting survival probability of Cas9-RNA bound states vs. time. **(a)** The survival probability vs. time for different DNA targets (colored circles) of the putative bound states (FRET>0.2) (double exponential fit in black). **(b)** The parameters, of the double

exponential fit which results in two characteristic bound state lifetimes i.e. long lived and transient binding with their characteristic amplitudes. Error bars represent s.d. for $n = 3$ ($n = 2$ for few sets).

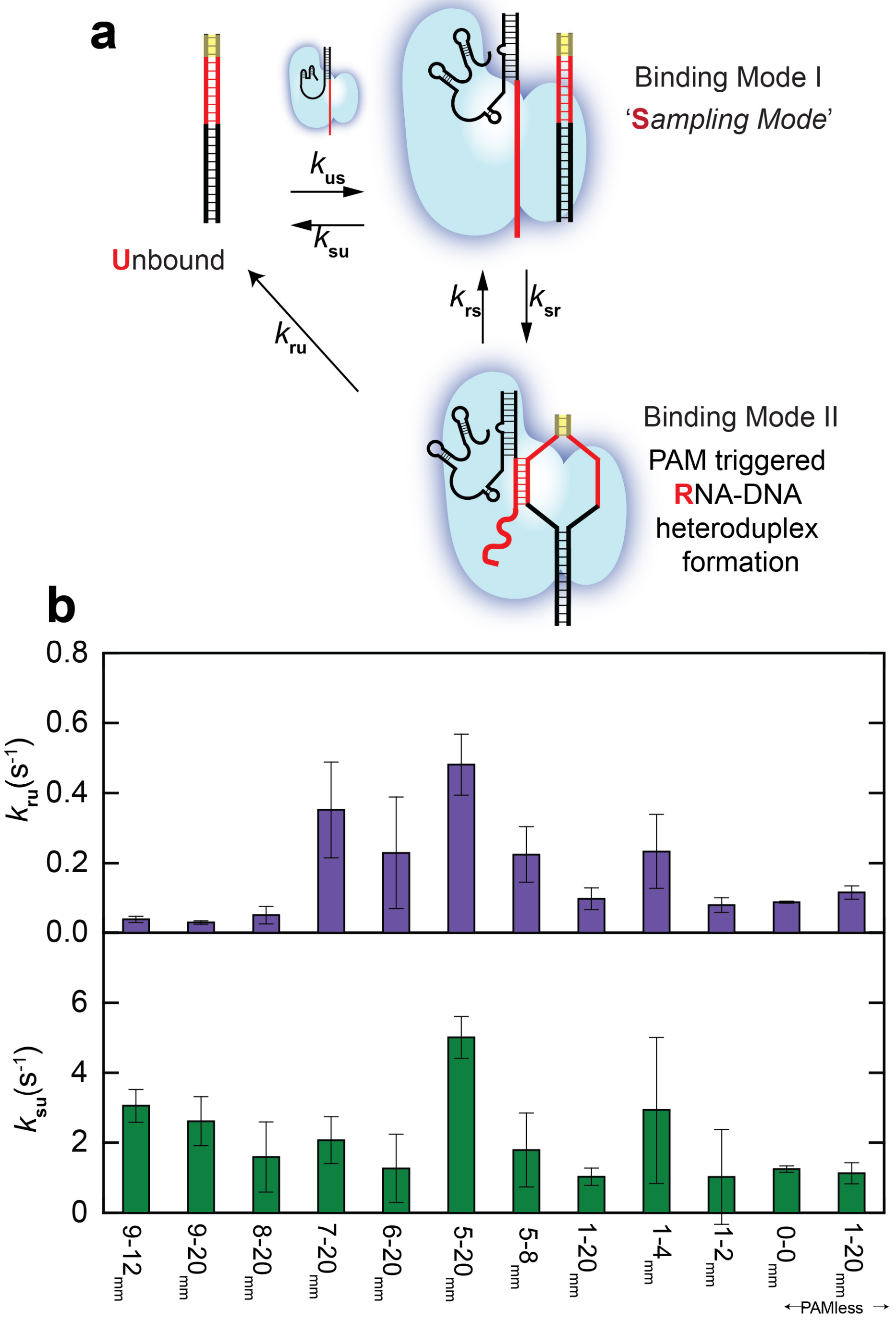

Supplementary Figure 12. Kinetic model describing the transitions between various states of Cas9-RNA DNA targeting.

(a) The kinetic model of Cas9-RNA and DNA interaction. Cas9-RNA has two binding modes

i.e. the sampling mode for PAM surveillance and the RNA-DNA heteroduplex mode which are referred to as **s** and **r**. Unbound DNA state is **u**. **(b)** Rates of transitions from RNA-DNA heteroduplex and sampling mode to unbound state for certain DNA targets including the ones without PAM. Error bars represent s.d. for n = 3 (n = 2 for few sets).

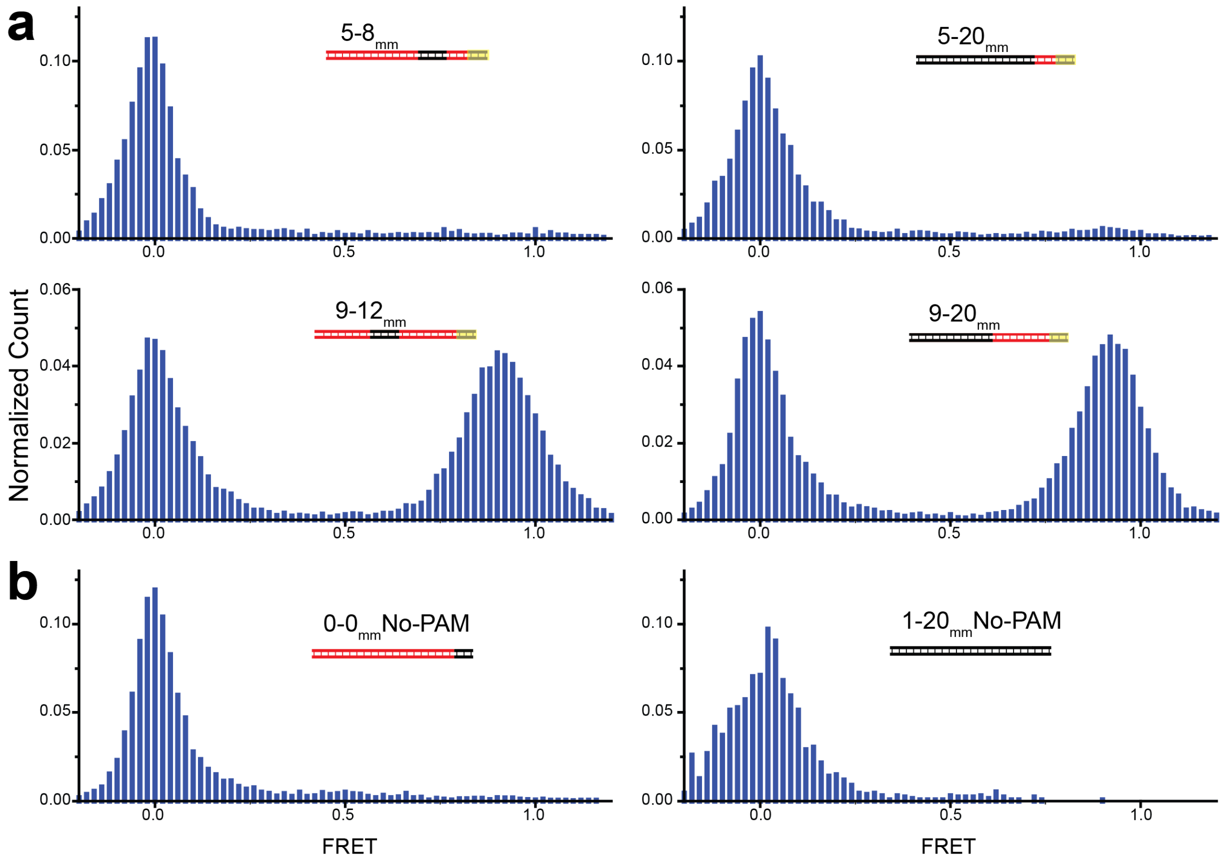

Supplementary Figure 13. FRET histograms for roadblock constructs and 'PAM-less' constructs.

(a) FRET histograms for DNA targets with and without mismatches beyond 4 bp roadblock starting from 8th or 5th bp from PAM. **(b)** FRET histograms for DNA with full sequence complementarity but no PAM (left), and for DNA with no sequence complementarity and no PAM. The number of molecules per histogram ranged from 297 to 3,053.

Supplementary Table 1: Different DNA targets used in this study for Cas9-RNA binding and RNA sequences for constituting Cas9-RNA

Description	DNA Sequences	Used in:
CognateSequence	20 nucleotide biotinylated adaptor for surface immobilization<br>5′-*-AACGCAACGTCGTCAGCTGTCT GCACAGCAGAAATCTCTGCTGACGCATAAAGATGAGACGCTGGAGTACAAACGTCAGCTTGCT-3′<br>3′-GCGTTGCAGCAGTCGACAGA-CGTGTCGTCTTTAGAGACGACTGCGTATTTCTACTCTGCGACCTCATGTTTGCAGTCGAACGA-5′	Fig. 1, 2<br>Supplementary Fig. 1-3, 6,7,9
17-20$_{mm}$	5′-*-AACGCAACGTCGTCAGCTGTCT GCACAGCAGAAATCTCTGCTCTGCCATAAAGATGAGACGCTGGAGTACAAACGTCAGCTTGCT-3′<br>3′-GCGTTGCAGCAGTCGACAGA-CGTGTCGTCTTTAGAGACGAGACGGTATTTCTACTCTGCGACCTCATGTTTGCAGTCGAACGA-5′	Fig. 2
13-20$_{mm}$	5′-*-AACGCAACGTCGTCAGCTGTCT GCACAGCAGAAATCTCTGCTCTGCGTATAAGATGAGACGCTGGAGTACAAACGTCAGCTTGCT-3′<br>3′-GCGTTGCAGCAGTCGACAGA-CGTGTCGTCTTTAGAGACGAGACGCATATTCTACTCTGCGACCTCATGTTTGCAGTCGAACGA-5′	Fig. 2
12-20$_{mm}$	5′-*-AACGCAACGTCGTCAGCTGTCT GCACAGCAGAAATCTCTGCTCTGCGTATTAGATGAGACGCTGGAGTACAAACGTCAGCTTGCT-3′<br>3′-GCGTTGCAGCAGTCGACAGA-CGTGTCGTCTTTAGAGACGAGACGCATAATCTACTCTGCGACCTCATGTTTGCAGTCGAACGA-5′	Fig. 2
11-20$_{mm}$	5′-*-AACGCAACGTCGTCAGCTGTCT GCACAGCAGAAATCTCTGCTCTGCGTATTTGATGAGACGCTGGAGTACAAACGTCAGCTTGCT-3′<br>3′-GCGTTGCAGCAGTCGACAGA-CGTGTCGTCTTTAGAGACGAGACGCATAAACTACTCTGCGACCTCATGTTTGCAGTCGAACGA-5′	Fig. 2
10-20$_{mm}$	5′-*-AACGCAACGTCGTCAGCTGTCT GCACAGCAGAAATCTCTGCTCTGCGTATTTCATGAGACGCTGGAGTACAAACGTCAGCTTGCT-3′<br>3′-GCGTTGCAGCAGTCGACAGA-CGTGTCGTCTTTAGAGACGAGACGCATAAAGTACTCTGCGACCTCATGTTTGCAGTCGAACGA-5′	Fig. 2<br>Supplementary Fig. 2, 7, 9
9-20$_{mm}$	5′-*-AACGCAACGTCGTCAGCTGTCT GCACAGCAGAAATCTCTGCTCTGCGTATTTCTTGAGACGCTGGAGTACAAACGTCAGCTTGCT-3′<br>3′-GCGTTGCAGCAGTCGACAGA-CGTGTCGTCTTTAGAGACGAGACGCATAAAGAACTCTGCGACCTCATGTTTGCAGTCGAACGA-5′	Fig. 2-3<br>Supplementary Fig. 4,7-9,11
8-20$_{mm}$	5′-*-AACGCAACGTCGTCAGCTGTCT GCACAGCAGAAATCTCTGCTCTGCGTATTTCTAGAGACGCTGGAGTACAAACGTCAGCTTGCT-3′<br>3′-GCGTTGCAGCAGTCGACAGA-CGTGTCGTCTTTAGAGACGAGACGCATAAAGATCTCTGCGACCTCATGTTTGCAGTCGAACGA-5′	Fig. 2-3<br>Supplementary Fig. 8,9
7-20$_{mm}$	5′-*-AACGCAACGTCGTCAGCTGTCT GCACAGCAGAAATCTCTGCTCTGCGTATTTCTACAGACGCTGGAGTACAAACGTCAGCTTGCT-3′<br>3′-GCGTTGCAGCAGTCGACAGA-CGTGTCGTCTTTAGAGACGAGACGCATAAAGATGTCTGCGACCTCATGTTTGCAGTCGAACGA-5′	Fig. 2-3<br>Supplementary Fig. 9
6-20$_{mm}$	5′-*-AACGCAACGTCGTCAGCTGTCT GCACAGCAGAAATCTCTGCTCTGCGTATTTCTACTGACGCTGGAGTACAAACGTCAGCTTGCT-3′<br>3′-GCGTTGCAGCAGTCGACAGA-CGTGTCGTCTTTAGAGACGAGACGCATAAAGATGACTGCGACCTCATGTTTGCAGTCGAACGA-5′	Fig. 2-3<br>Supplementary Fig. 9
5-20$_{mm}$	5′-*-AACGCAACGTCGTCAGCTGTCT GCACAGCAGAAATCTCTGCTCTGCGTATTTCTACTCACGCTGGAGTACAAACGTCAGCTTGCT-3′<br>3′-GCGTTGCAGCAGTCGACAGA-CGTGTCGTCTTTAGAGACGAGACGCATAAAGATGAGTGCGACCTCATGTTTGCAGTCGAACGA-5′	Fig. 2-3<br>Supplementary Fig. 4,5,8,9,11
1-20$_{mm}$	5′-*-AACGCAACGTCGTCAGCTGTCT GCACAGCAGAAATCTCTGCTCTGCGTATTTCTACTCTGCGTGGAGTACAAACGTCAGCTTGCT-3′<br>3′-GCGTTGCAGCAGTCGACAGA-CGTGTCGTCTTTAGAGACGAGACGCATAAAGATGAGACGCACCTCATGTTTGCAGTCGAACGA-5′	Fig. 1-3<br>Supplementary Fig. 9<br>(*Non-cognate in Fig.1)
1-2$_{mm}$	5′-*-AACGCAACGTCGTCAGCTGTCT GCACAGCAGAAATCTCTGCTGACGCATAAAGATGAGACCGTGGAGTACAAACGTCAGCTTGCT-3′<br>3′-GCGTTGCAGCAGTCGACAGA-CGTGTCGTCTTTAGAGACGACTGCGTATTTCTACTCTGGCACCTCATGTTTGCAGTCGAACGA-5′	Fig. 2-3<br>Supplementary Fig. 4, 9
1-4$_{mm}$	5′-*-AACGCAACGTCGTCAGCTGTCT GCACAGCAGAAATCTCTGCTGACGCATAAAGATGAGTGCGTGGAGTACAAACGTCAGCTTGCT-3′<br>3′-GCGTTGCAGCAGTCGACAGA-CGTGTCGTCTTTAGAGACGACTGCGTATTTCTACTCACGCACCTCATGTTTGCAGTCGAACGA-5′	Fig. 2-3<br>Supplementary Fig. 4,9
9-12$_{mm}$	5′-*-AACGCAACGTCGTCAGCTGTCT GCACAGCAGAAATCTCTGCTGACGCATATTCTTGAGACGCTGGAGTACAAACGTCAGCTTGCT-3′<br>3′-GCGTTGCAGCAGTCGACAGA-CGTGTCGTCTTTAGAGACGACTGCGTATAAGAACTCTGCGACCTCATGTTTGCAGTCGAACGA-5′	Fig. 2-3<br>Supplementary Fig. 7,9,11
5-8$_{mm}$	5′-*-AACGCAACGTCGTCAGCTGTCT GCACAGCAGAAATCTCTGCTGACGCATAAAGAACTCACGCTGGAGTACAAACGTCAGCTTGCT-3′<br>3′-GCGTTGCAGCAGTCGACAGA-CGTGTCGTCTTTAGAGACGACTGCGTATTTCTTGAGTGCGACCTCATGTTTGCAGTCGAACGA-5′	Fig. 2-3<br>Supplementary Fig. 9,11
0-0$_{mm}$ No-PAM	5′-*-AACGCAACGTCGTCAGCTGTCT GCACAGCAGAAATCTCTGCTGACGCATAAAGATGAGACGCATAAGTACAAACGTCAGCTTGCT-3′<br>3′-GCGTTGCAGCAGTCGACAGA-CGTGTCGTCTTTAGAGACGACTGCGTATTTCTACTCTGCGTATTCATGTTTGCAGTCGAACGA-5′	Supplementary Fig. 4,6,9,11
1-20$_{mm}$ No-PAM	5′-*-AACGCAACGTCGTCAGCTGTCT GCACAGCAGAAATCTCTGCTCTGCGTATTTCTACTCTGCGATAAGTACAAACGTCAGCTTGCT-3′<br>3′-GCGTTGCAGCAGTCGACAGA-CGTGTCGTCTTTAGAGACGAGACGCATAAAGATGAGACGCTATTCATGTTTGCAGTCGAACGA-5′	Supplementary Fig. 9-11

Description	RNA Sequences
crRNA	5′-GACGCAUAAAGAUGAGACGCGUUUUAGAGCUAUGCUGUUUUG-3′
tracrRNA	5′-GGACAGCAUAGCAAGUUAAAAUAAGGCUAGUCCGUUAUCAACUUGAAAAAGUGGCACCGAGUCGGUGCUUUUU-3′

Biotin   Protospacer Adjacent Motif (PAM)   Thymine modification for Cy3 and Cy5 labeling

DNA sequences complementary to guide RNA are shown in red (Cognate).
RNA sequences complementary to the protospacer in a cognate DNA target are shown in red (Cognate).

Supplementary Table 1: Different DNA targets used in this study for Cas9-RNA binding and RNA sequences for constituting Cas9-RNA

Description	DNA sequences
Cognate Sequence	5′-*-AACGCAACGTCGTCAGCTGTCT GCACAGCAGAAATCTCTGCTGACGCATAAAGATGAGACGCTGGAGTACAAACGTCAGCTTGCT-3′<br>3′-GCGTTGCAGCAGTCGACAGA-CGTGTCGTCTTTAGAGACGACTGCGTATTTCTACTCTGCGACCTCATGTTTGCAGTCGAACGA-5′
17-20$_{mm}$	5′-*-AACGCAACGTCGTCAGCTGTCT GCACAGCAGAAATCTCTGCTCTGCCATAAAGATGAGACGCTGGAGTACAAACGTCAGCTTGCT-3′<br>3′-GCGTTGCAGCAGTCGACAGA-CGTGTCGTCTTTAGAGACGAGACGGTATTTCTACTCTGCGACCTCATGTTTGCAGTCGAACGA-5′
13-20$_{mm}$	5′-*-AACGCAACGTCGTCAGCTGTCT GCACAGCAGAAATCTCTGCTCTGCGTATAAGATGAGACGCTGGAGTACAAACGTCAGCTTGCT-3′<br>3′ -GCGTTGCAGCAGTCGACAGA-CGTGTCGTCTTTAGAGACGAGACGCATATTCTACTCTGCGACCTCATGTTTGCAGTCGAACGA-5′
12-20$_{mm}$	5′-*-AACGCAACGTCGTCAGCTGTCT GCACAGCAGAAATCTCTGCTCTGCGTATTAGATGAGACGCTGGAGTACAAACGTCAGCTTGCT-3′<br>3′-GCGTTGCAGCAGTCGACAGA-CGTGTCGTCTTTAGAGACGAGACGCATAATCTACTCTGCGACCTCATGTTTGCAGTCGAACGA-5′
11-20$_{mm}$	5′-*-AACGCAACGTCGTCAGCTGTCT GCACAGCAGAAATCTCTGCTCTGCGTATTTGATGAGACGCTGGAGTACAAACGTCAGCTTGCT-3′<br>3′ -GCGTTGCAGCAGTCGACAGA-CGTGTCGTCTTTAGAGACGAGACGCATAAACTACTCTGCGACCTCATGTTTGCAGTCGAACGA-5′
10-20$_{mm}$	5′-*-AACGCAACGTCGTCAGCTGTCT GCACAGCAGAAATCTCTGCTCTGCGTATTTCATGAGACGCTGGAGTACAAACGTCAGCTTGCT-3′<br>3′- GCGTTGCAGCAGTCGACAGA-CGTGTCGTCTTTAGAGACGAGACGCATAAAGTACTCTGCGACCTCATGTTTGCAGTCGAACGA-5′
9-20$_{mm}$	5′-*-AACGCAACGTCGTCAGCTGTCT GCACAGCAGAAATCTCTGCTCTGCGTATTTCTTGAGACGCTGGAGTACAAACGTCAGCTTGCT-3′<br>3′-GCGTTGCAGCAGTCGACAGA-CGTGTCGTCTTTAGAGACGAGACGCATAAAGAACTCTGCGACCTCATGTTTGCAGTCGAACGA-5′
8-20$_{mm}$	5′-*-AACGCAACGTCGTCAGCTGTCT GCACAGCAGAAATCTCTGCTCTGCGTATTTCTAGAGACGCTGGAGTACAAACGTCAGCTTGCT-3′<br>3′-GCGTTGCAGCAGTCGACAGA-CGTGTCGTCTTTAGAGACGAGACGCATAAAGATCTCTGCGACCTCATGTTTGCAGTCGAACGA-5′
7-20$_{mm}$	5′-*-AACGCAACGTCGTCAGCTGTCT GCACAGCAGAAATCTCTGCTCTGCGTATTTCTACAGACGCTGGAGTACAAACGTCAGCTTGCT-3′<br>3′-GCGTTGCAGCAGTCGACAGA-CGTGTCGTCTTTAGAGACGAGACGCATAAAGATGTCTGCGACCTCATGTTTGCAGTCGAACGA-5′
6-20$_{mm}$	5′-*-AACGCAACGTCGTCAGCTGTCT GCACAGCAGAAATCTCTGCTCTGCGTATTTCTACTGACGCTGGAGTACAAACGTCAGCTTGCT-3′<br>3′-GCGTTGCAGCAGTCGACAGA-CGTGTCGTCTTTAGAGACGAGACGCATAAAGATGACTGCGACCTCATGTTTGCAGTCGAACGA-5′
5-20$_{mm}$	5′-*-AACGCAACGTCGTCAGCTGTCT GCACAGCAGAAATCTCTGCTCTGCGTATTTCTACTCACGCTGGAGTACAAACGTCAGCTTGCT-3′<br>3′-GCGTTGCAGCAGTCGACAGA-CGTGTCGTCTTTAGAGACGAGACGCATAAAGATGAGTGCGACCTCATGTTTGCAGTCGAACGA-5′
1-20$_{mm}$	5′-*-AACGCAACGTCGTCAGCTGTCT GCACAGCAGAAATCTCTGCTCTGCGTATTTCTACTCTGCGTGGAGTACAAACGTCAGCTTGCT-3′<br>3′-GCGTTGCAGCAGTCGACAGA-CGTGTCGTCTTTAGAGACGAGACGCATAAAGATGAGACGCACCTCATGTTTGCAGTCGAACGA-5′
1-2$_{mm}$	5′-*-AACGCAACGTCGTCAGCTGTCT GCACAGCAGAAATCTCTGCTGACGCATAAAGATGAGACCGTGGAGTACAAACGTCAGCTTGCT-3′<br>3′-GCGTTGCAGCAGTCGACAGA-CGTGTCGTCTTTAGAGACGACTGCGTATTTCTACTCTGGCACCTCATGTTTGCAGTCGAACGA-5′
1-4$_{mm}$	5′-*-AACGCAACGTCGTCAGCTGTCT GCACAGCAGAAATCTCTGCTGACGCATAAAGATGAGTGCGTGGAGTACAAACGTCAGCTTGCT-3′<br>3′-GCGTTGCAGCAGTCGACAGA-CGTGTCGTCTTTAGAGACGACTGCGTATTTCTACTCACGCACCTCATGTTTGCAGTCGAACGA-5′
9-12$_{mm}$	5′-*-AACGCAACGTCGTCAGCTGTCT GCACAGCAGAAATCTCTGCTGACGCATATTCTTGAGACGCTGGAGTACAAACGTCAGCTTGCT-3′<br>3′-GCGTTGCAGCAGTCGACAGA-CGTGTCGTCTTTAGAGACGACTGCGTATAAGAACTCTGCGACCTCATGTTTGCAGTCGAACGA-5′

5-8$_{mm}$	5′-*-AACGCAACGTCGTCAGCTGTCT GCACAGCAGAAATCTCTGCT**GACGCATAAAGA**ACTC**ACGCTGG**AGTACAAACGTCAGCTTGCT-3′<br>3′-GCGTTGCAGCAGTCGACAGA-CGTGTCGTCTTTAGAGACGA**CTGCGTATTTCT**TGAG**TGCG**ACCTCATGTTTGCAGTCGAACGA-5′
0-0$_{mm}$ (NoPAM)	5′-*-AACGCAACGTCGTCAGCTGTCT GCACAGCAGAAATCTCTGCT**GACGCATAAAGATGAGACGCATA**AGTACAAACGTCAGCTTGCT-3′<br>3′- GCGTTGCAGCAGTCGACAGA-CGTGTCGTCTTTAGAGACGA**CTGCGTATTTCTACTCTGCG**TATTCATGTTTGCAGTCGAACGA-5′
1-20$_{mm}$ NoPAM	5′-*-AACGCAACGTCGTCAGCTGTCT GCACAGCAGAAATCTCTGCTCTGCGTATTTCTACTCTGCG**ATA**AGTACAAACGTCAGCTTGCT-3′<br>3′-GCGTTGCAGCAGTCGACAGA-CGTGTCGTCTTTAGAGACGAGACGCATAAAGATGAGACGCTATTCATGTTTGCAGTCGAACGA-5′
**Description**	**RNA sequences**
crRNA	5′-GACGCAUAAAGAUGAGACGCGUUUUAGAGCUAUGCUGUUUUG-3′
tracrRNA	5′-GGACAGCAUAGCAAGUUAAAAUAAGGCUAGUCCGUUAUCAACUUGAAAAAGUGGCACCGAGUCGGUGCUUUUU-3′

Biotin *

DNA sequences complementary to guide RNA are shown in red (cognate).

Protospacer adjacent motif (PAM) are highlighted in yellow and thymine modifications for Cy3 (DNA targets) and Cy5 labeling (guide-RNA) are highlighted in green and red respectively.

Supplementary Table 2: The abundance count of the sequence of the different DNA targets in human genome (GRCh38.p6) along with its probabilistic count.

Description	DNA Sequences	Actual Count	Probablistic Count
CognateSequence	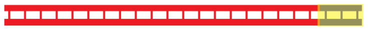	0	0
$17\text{-}20_{mm}$	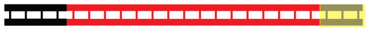	0	0
$13\text{-}20_{mm}$	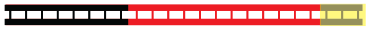	8	24
$12\text{-}20_{mm}$	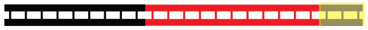	35	96
$11\text{-}20_{mm}$	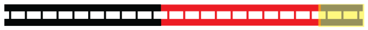	99	382
$10\text{-}20_{mm}$	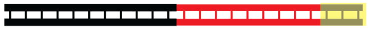	211	763
$9\text{-}20_{mm}$	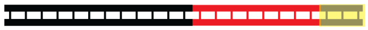	1146	1526
$8\text{-}20_{mm}$	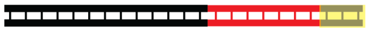	5936	24414
$7\text{-}20_{mm}$	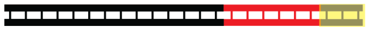	18370	48828
$6\text{-}20_{mm}$	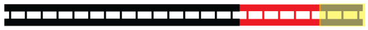	59365	97656
$5\text{-}20_{mm}$	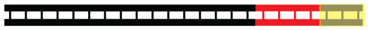	222480	1562500

Supplementary Table 2: The abundance count of the sequence of the different DNA targets in human genome (GRCh38.p6) along with its probabilistic count.

Description	Actual Count	Probabilistic Count
Cognate Sequence	0	0
$17\text{-}20_{mm}$	0	0
$13\text{-}20_{mm}$	8	24
$12\text{-}20_{mm}$	35	96
$11\text{-}20_{mm}$	99	382
$10\text{-}20_{mm}$	211	763
$9\text{-}20_{mm}$	1146	1526
$8\text{-}20_{mm}$	5936	24414
$7\text{-}20_{mm}$	18370	48828
$6\text{-}20_{mm}$	59365	97656
$5\text{-}20_{mm}$	222480	1562500

Supplementary References

1. Anders, C., Niewoehner, O., Duerst, A. & Jinek, M. Structural basis of PAM-dependent target DNA recognition by the Cas9 endonuclease. *Nature* **513**, 569-573 (2014).
2. Sternberg, S.H., Redding, S., Jinek, M., Greene, E.C. & Doudna, J.A. DNA interrogation by the CRISPR RNA-guided endonuclease Cas9. *Nature* **507**, 62-67 (2014).
3. McKinney, S.A., Joo, C. & Ha, T. Analysis of Single-Molecule FRET Trajectories Using Hidden Markov Modeling. *Biophysical Journal* **91**, 1941-1951 (2006).
